# The Redox State of Cytochrome C Modulates Resistance to Methotrexate in Human MCF7 Breast Cancer Cells

**DOI:** 10.1371/journal.pone.0063276

**Published:** 2013-05-13

**Authors:** Susana Barros, Núria Mencia, Laura Rodríguez, Carlota Oleaga, Conceição Santos, Verónique Noé, Carlos J. Ciudad

**Affiliations:** 1 Department of Biochemistry and Molecular Biology, School of Pharmacy, University of Barcelona, Barcelona, Spain; 2 Department of Biology, CESAM, University of Aveiro, Campus Universitário de Santiago, Aveiro, Portugal; The Ohio State University, United States of America

## Abstract

**Background:**

Methotrexate is a chemotherapeutic agent used to treat a variety of cancers. However, the occurrence of resistance limits its effectiveness. Cytochrome c in its reduced state is less capable of triggering the apoptotic cascade. Thus, we set up to study the relationship among redox state of cytochrome c, apoptosis and the development of resistance to methotrexate in MCF7 human breast cancer cells.

**Results:**

Cell incubation with cytochrome c-reducing agents, such as tetramethylphenylenediamine, ascorbate or reduced glutathione, decreased the mortality and apoptosis triggered by methotrexate. Conversely, depletion of glutathione increased the apoptotic action of methotrexate, showing an involvement of cytochrome c redox state in methotrexate-induced apoptosis. Methotrexate-resistant MCF7 cells showed increased levels of endogenous reduced glutathione and a higher capability to reduce exogenous cytochrome c. Using functional genomics we detected the overexpression of GSTM1 and GSTM4 in methotrexate-resistant MCF7 breast cancer cells, and determined that methotrexate was susceptible of glutathionylation by GSTs. The inhibition of these GSTM isoforms caused an increase in methotrexate cytotoxicity in sensitive and resistant cells.

**Conclusions:**

We conclude that overexpression of specific GSTMs, GSTM1 and GSTM4, together with increased endogenous reduced glutathione levels help to maintain a more reduced state of cytochrome c which, in turn, would decrease apoptosis, thus contributing to methotrexate resistance in human MCF7 breast cancer cells.

## Introduction

Methotrexate (MTX) is a chemotherapeutic agent widely used, alone or in combination with other chemotherapeutic agents, for the treatment of a range of cancers, such as breast cancer, osteosarcoma, head and neck cancer, lymphoma and acute lymphoblastic leukemia [1]. As a structural analogue of folic acid, MTX is a high affinity inhibitor of dihydrofolate reductase (DHFR) by competing with dihydrofolate for the active site. DHFR catalyzes the NADPH-dependent reduction of dihydrofolate to tetrahydrofolate involved in the biosynthesis of thymidylate, hypoxantine and glycine, needed for DNA synthesis [2]–[4]. Once DHFR is inhibited by MTX, there is suppression of DNA synthesis and cell proliferation is affected. However, the main drawback of using MTX in cancer therapy is the occurrence of resistance upon treatment, thus compromising its effectiveness. Several MTX resistance mechanisms had been described such as gene amplification of the *dhfr locus* [5], [6], deficiency in MTX transport [7], [8] or MTX polyglutamation [9], expression of the MDR phenotype and mutations of the target (DHFR protein) [10]. Altered gene or miRNA expression also contribute to MTX resistance such as increases in AKR1C1 [11], S100A4 [12], caveolin-1, enolase-2, PRKCA [13], DKK1, EEF1A1, and UGT1A family [14], [15], and the decrease of E-Cadherin [13] or miR-224 [16].

Reduced glutathione (GSH) and Glutathione S-transferases (GSTs) have been implicated in the development of drug resistance in cancer chemotherapy [17], [18]. The GSTs enzymatic family belongs to a Phase II detoxification program functioning as a cellular protection from attack by reactive electrophiles associated to environmental stresses and drugs [19]. This family is mainly responsible for the conjugation of GSH to electrophilic compounds and includes three main types, cytosolic, mitochondrial and membrane-bound microsomal. Cytosolic GSTs are divided into seven classes: alpha (A), Mu (M), Omega (O), Pi (P), Sigma (S), Theta (T) and Zeta (Z) [20]–[23]. GSTs are thought to be involved in the development of drug resistance via direct detoxification or by regulation of the MAP kinase pathway, specifically JNK-pathway as reviewed in [19].

Cytochrome c (cyt c) is a heme-protein bound to the mitochondrial inner membrane by an interaction with the anionic phospholipid cardiolipin, which keeps cytochrome c in its proper location and prevents its release to the cytosol [24]. Under physiological conditions, cytochrome c is responsible for the electron transfer between complexes III and IV of the mitochondrial electron transport chain whereas under oxidative stress, the peroxidase activity of cytochrome c is activated, cardiolipin becomes peroxidized, and loses its affinity for cytochrome c allowing its release to the cytosol [25], [26]. Once in the cytosol, cytochrome c can only induce apoptosis in its oxidized form [27]–[31]. The presence of high levels of cytosolic GSH holds the released cytochrome c inactive in a reduced state, thus preventing the progression of the apoptotic cascade [32], [33].

In this study, we investigated the effect of the reduction state of cytochrome c on MTX sensitivity and apoptosis and its relationship with the different GSTs overexpressed in MCF7 breast cancer cells resistant to MTX, to evaluate a possible connection between GSTs and GSH in the reduction state of cytochrome c and the development of MTX resistance.

## Materials and Methods

### Cell lines and cell culture

The following human cell lines were used: MCF7, MDA-MB-468 and 10^−6^ M MTX-resistant MCF7 cells from breast cancer; SaOs-2 and 10^−6^ M MTX-resistant SaOs-2 cells from osteosarcoma; and HT-29 and CaCo-2 cells from colon cancer. Resistant cells were obtained previously in the laboratory upon incubation with stepwise concentrations of MTX (Almirall, Barcelona, Spain) as described in [11]. In all experimental procedures, cells were grown in Ham's F12 medium lacking the final products of DHFR activity, glycine, hypoxanthine and thymidine (-GHT), and supplemented with 7% v/v dialyzed fetal bovine serum (GIBCO, Life Technologies, Madrid, Spain), 14 mM sodium bicarbonate (1.176 g/l), penicillin G (100 U/ml) and streptomycin (100 mg/l). Cells were maintained at 37°C in a humidified atmosphere of 5% CO_2_ in air. Before reaching 70% confluence, cells were sub-cultured by treatment with 0.05% trypsin in PBS 1×. All these components were purchased from Sigma-Aldrich (Madrid, Spain).

### Oxygen consumption assay

Cellular oxygen consumption was monitored polarographically with a Clark-type oxygen electrode using Hansatech Oxygraph Measurement System (Hansatech, Norfolk, UK). The assay was performed using 6×10^4^ cells/ml in the presence or in the absence of 5 µM TMPD or 300 µM ASC in 1 ml of PBS (pH 7.4) as experimental medium at 37°C. Oxygen consumption was measured during 5 min for each condition and determined by the slope calculated directly by the Oxygraph Plus Software.

### Cell viability assay

Cell viability was assessed by the MTT (3-(4,5-Dimethylthiazol-2-yl)-2,5-diphenyltetrazolium bromide) assay [34] in 6-well dishes. Cells were incubated with 500 µg of MTT and 0.270 mg of sodium succinate (Sigma-Aldrich, Madrid, Spain) and allowed to react for 2 h at 37°C. The medium was removed and 1 ml of the solubilization reagent (0,57% acetic acid and 10% SDS in DMSO (dimethyl sulfoxide)) was added (Applichem, Ecogen, Barcelona, Spain). Cell viability was measured at 570 nm in a WPA S2100 Diode Array Spectrophotometer. The results were expressed as percentage of cell survival relative to the control (untreated cells).

### Microarrays

Gene expression was analyzed by hybridization to GeneChip® Human Genome U133 PLUS 2.0 microarrays from Affymetrix, containing 54,675 transcripts and variants. Total RNA for cDNA arrays was prepared from triplicate samples from both control and resistant cells using the RNAeasy Mini kit (Qiagen, Madrid, Spain) following the recommendations of the manufacturer. The integrity of the RNA species was checked using the Bioanalyzer 2100 system (Agilent, Madrid, Spain). Labeling, hybridization and detection were carried out following the manufacturer's specifications.

### Microarrays data analyses

Quantification was carried out with GeneSpring GX 12.0 software (Agilent, Madrid, Spain), which allows multi-filter comparisons using data from different experiments to perform the normalization, generation of lists and the functional classification of the differentially expressed genes. The input data was subjected to preprocess baseline transformation using the RMA summarization algorithm using the median of control samples. After grouping the triplicates of each experimental condition, lists of differentially expressed genes could be generated by using volcano plot analysis. T-test unpaired was applied using asymptotic *p*-value computation and multiple testing correction of Benjamini-Hochberg false discovery rate, FDR. The expression of each gene was reported as the ratio of the value obtained for the resistant condition relative to the control condition after normalization and statistical analysis of the data. The corrected *p*-value cut-off applied was of <0.05; then the output of this statistical analysis was filtered by fold expression, selecting specifically those genes that had a differential expression of at least 2-fold.

### RT-Real-Time PCR

Total RNA was extracted using the Ultraspec™ RNA reagent (Biotecx, Ecogen, Barcelona, Spain) following the manufacturer's instructions. Complementary DNA (cDNA) was synthetized in a total volume of 20 µl by mixing 1 µg of total RNA, 125 ng of random hexamers (Roche, Mannheim, Germany), in the presence of 75 mM KCl, 3 mM MgCl_2_, 50 mM Tris-HCl buffer, pH 8.3, 10 mM dithiothreitol (Invitrogen, Life Technologies, Madrid, Spain), 20 units of RNAsin (Promega, Madrid, Spain), 0.5 mM dNTPs (Applichem, Ecogen, Barcelona, Spain) and 200 units of MLV-reverse transcriptase (Invitrogen, Life Technologies, Madrid, Spain). The reaction mix was incubated at 37°C for 1 h and the cDNA product was used for subsequent amplification.

Gene expression levels were quantified by SYBR Green RT-Real Time PCR reaction in a final volume of 20 µl with specific forward and reverse primers, using the StepOnePlus™ detection system (Applied Biosystems, Life Technologies, Madrid, Spain). The sequences of the forward and reverse primers (Sigma-Aldrich, Madrid, Spain) are given in Table 1.

**Table 1:** Primer sequences.

Gene	Forward (5′-3′)	Reverse (5′-3′)
**GSTM1**	TGAAGCCTCAGCTACCCACT	AACCAGTCAATGCTGCTCCT
**GSTM4**	TTTCCTCGCCTATGATGTCC	GCTGAGTATGGGCTCCTCAC
**HPRT**	TGCTCGAGATGTGATGAAGG	TCCCCTGTTGACTGGTCATT

The sequences for the forward and reverse primer for detection of GSTM1, GSTM4 and HPRT mRNA levels used for RT-Real Time PCR are given.

Changes in gene expression were calculated using the quantitative ΔΔCt method and normalized against Hypoxanthine-phosphoribosyl transferase (HPRT) in each sample.

### GSTM1 and GSTM4 protein levels

MCF7 cells, either sensitive or MTX-resistant, were harvested from confluent dishes and total extracts were prepared according to [14]. Total extracts (80 µg) were resolved on a 15% SDS-PAGE (AppliChem, Ecogen, Barcelona, Spain) and transferred to PVDF membranes (Immobilon P, Millipore, Madrid, Spain) using a semidry electroblotter. The membranes were probed with anti-GSTμ antibody (FL-218, sc-292368) (Santa Cruz Biotechnology Inc, Heidelberg, Germany) 1∶200 dilution, OVN at 4°C. Signals were detected using ImageQuant LAS 4000 Mini Technology (Amersham, GE Healthcare Life Sciences, Barcelona, Spain) with rabbit secondary horseradish peroxidase–conjugated antibody (P0399) (Dako, Barcelona, Spain) 1∶5000 dilution, for 1 h at room temperature. To normalize the results blots were reprobed with an antibody against tubulin (Cp06) (Calbiochem, Millipore, Merck, Madrid, Spain) 1∶400 dilution, OVN at 4°C and detected with anti-mouse (NIF 824) (Amersham, GE Healthcare Life Sciences, Barcelona, Spain) 1∶2500 dilution, for 1 h at room temperature.

### Inhibition of GSTM1 and GSTM4 expression levels

GST expression was inhibited by specific PPRH-hairpins (hp), a new class of DNA-hairpin molecules able to silence gene expression [35], [36]. The Triplex-Forming Oligonucleotide Target Sequence Search software (M.D. Anderson Cancer Center, Houston, TX) (spi.mdanderson.org/tfo/) was used to design the hairpins. BLAST analyses were performed to check for the specificity of each sequence. Cells were plated in 6-well dishes in 1 ml of medium the night before transfection and each hairpin was mixed with N-[1-(2,3-dioleoyloxy)propyl]-N,N,N-trimethylammonium methylsulfate (DOTAP) (Roche, Mannheim, Germany) at the appropriate oligonucleotide–DOTAP molar ratio (1∶100) for 15 min at RT before lipofecting the cells. The sequences of the hairpins (Sigma-Aldrich, Madrid, Spain) are listed in Table 2.

**Table 2:** Hairpin sequences.

Hairpin	Sequence (5′-3′)
**hpGSTM1**	GAAGGGGAGGGAAGAGAGAAGTTTTTGAAGAGAGAAGGGAGGGGAAG
**hpGSTM4**	GGAGAAGAAGAAAAGGGGGAAGTTTTTGAAGGGGGAAAAGAAGAAGAGG
**hpSC**	AAGAGAAAAAGAGAAAGAAGAGAGGGTTTTTGGGAGAGAAGAAAGAGAAAAAGAGAA
**hpGSTA4**	AAGGGAAGGGAGGAGGAAGAAAAGTTTTTGAAAAGAAGGAGGAGGGAAGGGAA

The sequences for the polypurine reverse Hoogsteen hairpins used for specific inhibition of GSTM1, GSTM4 or GSTA4 as well as the scrambled negative control are given.

For RNA determination, cells were transfected and total RNA was extracted 30 h later. Gene expression was quantified as described above.

For viability assays, cells were incubated with each hairpin for 24 h before MTX treatment. Survival was determined after 3 days in sensitive MCF7 cells or 6 days in resistant MCF7 and SaOs-2 cells.

### Apoptosis

Apoptosis was determined by the Rhodamine 123/Propidium Iodide (PI) assay. Cells (6×10^4^) were plated in 6-well dishes, and treated with the different agents (TMPD, ascorbate, veratridine) alone or in combination with MTX for the indicated times and concentrations. Then cells were incubated for 30 min with 5 µl of Rhodamine 123 (1 µg/µl). All the cells in each well were harvested and centrifuged at 800× *g* for 5 min. The cell pellet was washed twice with 1 ml of PBS 1×+1%BSA solution and resuspended in 500 µl of PBS 1×+BSA 1% solution containing 0.5 µl PI (5 µg/µl). The entire procedure was performed at 4°C. All these reagents were purchased from Sigma-Aldrich (Madrid, Spain). Samples were analyzed by flow cytometry in the Coulter Epics XL™ cytometer (Beckman, Barcelona, Spain) at an excitation wavelength of 488 nm by reading the fluorescence of rhodamine123 at 525 nm. Cells that were negative for both rhodamine123 and PI were counted as the apoptotic population. Summit v4.3 software was used to analyze the data.

### GSH endogenous levels

Endogenous GSH levels were determined using the Glutathione Assay Kit, Fluorimetric (Sigma-Aldrich®) based on a fluorimetric reaction catalyzed by GSTs between monochlorobimane (MCB), a thiol probe, and GSH. Briefly, the assay was performed with 6×10^4^ sensitive and resistant MCF7 cells and the formation of the fluorescent adduct GSH-monochlorobimane was monitored at 390 nm for excitation and 478 nm for emission during 1 h.

### Exogenous cyt c reduction by cytoplasmic cell extracts

Cytoplasmic cell extracts were obtained from MCF7 cells. Cells were collected in ice-cold PBS by scraping and centrifuged at 1,500× *g* for 10 min. The cell pellet was resuspended in 3 ml of lysing buffer prepared according to Borutaite&Brown [29] and homogenized in Glass/Teflon Potter Elvehjem homogenizer (20 strokes). The homogenate was further centrifuged in the same conditions as above and the supernatant was further centrifuged at 22,000× *g* for 30 min and the resulting supernatant corresponds to the cytoplasmic extract. The entire procedure was performed at 4°C. The reduction of exogenous cytochrome c by cytoplasmic extracts (100 µg/ml of total protein) was followed spectrophotometrically. The analysis measured the absorbance spectra between 500 and 600 nm wavelengths after incubation for 15 min at 37°C of exogenous cytochrome c (10 µM) with cytoplasmic cell extracts from sensitive or resistant MCF7 cells. Reduction level of cytochrome c was expressed as absorbance at 550 nm minus absorbance at 535 nm and was normalized to the protein of cytosolic extract used.

### In vitro Glutathionylation

The glutathionylation of MTX catalyzed by GSTs was determined *in vitro* and in cell free extracts. *In vitro*, the reaction was performed by incubating MCB (100 µM), GSH (30 µM) and GST (0.25 U) in the absence or the presence of different concentrations of MTX in 0.1 M potassium phosphate buffer pH 7.0 [37]. The reaction was performed at 37°C for 15 min and the fluorescence corresponding to the MCB-GSH adduct was measured as described above.

In cell free extracts, the reaction was performed as follows. Parental or MCF7 MTX-resistant cells (4×10^5^) were harvested and washed twice with ice-cold PBS 1×. The pelleted cells were then resuspended in 100 µl of PBS 1×-1% Triton X-100, kept on ice for 15 minutes and centrifuged at 15,000× *g* for 15 minutes (4°C). The corresponding supernatant was collected (300 µg) and used for the glutathionylation reaction in the absence or in the presence of MTX for 15 min at 37°C.

MCB, reduced glutathione and GST were purchased from Sigma-Aldrich (Madrid, Spain). MCB and GSH were resuspended in DMSO and GST was resuspended at a concentration 0.25 U/µl in 0.01 M potassium phosphate pH 7.0 and 30% glycerol buffer.

### Statistical analysis

Data are presented as the mean ± SE for at least three different experiments. Analyses were performed using SPSS v.18.3 software. Differences with *p*-value<0.05 were considered significant.

## Results and Discussion

### Effect of TMPD and ascorbate on the cytotoxicity produced by methotrexate

Tetramethylphenylenediamine (TMPD) and ascorbate (ASC) have been described as external reductants of cytochrome c both in cytosol and mitochondria [29], [38], [39]. In this direction, we used both agents to study the role of the reduced state of cytochrome c in the sensitivity to methotrexate.

**Figure 1:**
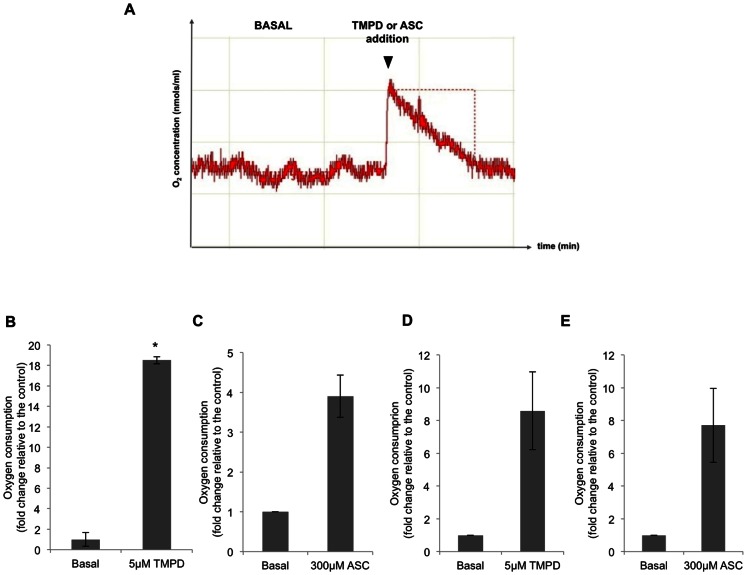
O_2_ consumption analysis upon treatment with TMPD or ascorbate (ASC). A) Representative image of changes in O_2_ concentration over time in cell extracts under basal conditions and after treatment with TMPD or ASC. The basal consumption rate in MCF7 cells (B–C) was 0.0335 nmol O_2_/ml/min and 0.047 nmol O_2_/ml/min in SaOs-2 cells (D–E). The results are expressed as the ratio of O_2_ consumption rate in each condition relative to basal consumption levels and represent the mean value ± SE of three independent experiments. * *p*<0.05.

First, the reduction of cytochrome c by TMPD or ASC treatment in MCF7 cells was confirmed by determining the changes in O_2_ consumption using an oxygraph upon treatment with these chemical reagents. One of the classic end-points to analyze mitochondrial function is to assess the changes in oxygen consumption since O_2_ is the ultimate electron acceptor [40]. This method is commonly used [39], [41]–[44], it calculates the variation of O_2_ concentration over time and offers the unique advantage of being able to add other components during the experiment. The slope of the graph represents the O_2_ consumption rate. As shown in figure 1, TMPD and ASC addition increased O_2_ consumption in MCF7 cells by 18.5 and 3.9 fold, respectively (Figure 1, B and C). In addition, the oxygen consumption rate was also determined in SaOs-2 cells upon addition of the 2 reducing agents, TMPD and ASC, causing an increase of 8.6 and 7.7 fold, respectively (Figure 1, D and E).

**Figure 2:**
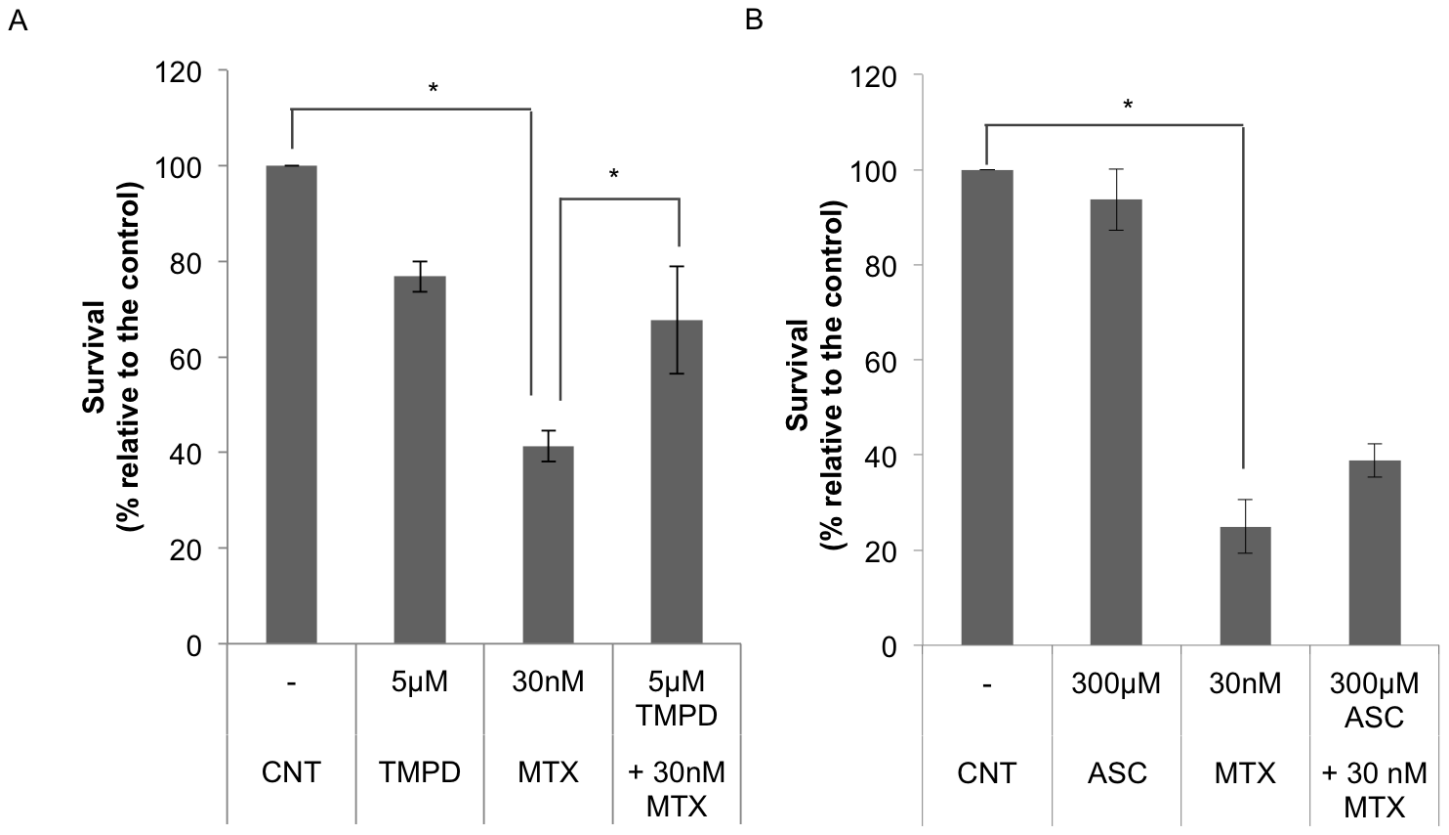
Effect of TMPD or ascorbate (ASC) in combination with MTX on cell viability. Cells (6×10^4^) were incubated in 1 ml of medium with TMPD (A) or ASC (B) either alone or in combination with MTX at the indicated concentrations, for 3 or 6 days, respectively, and cell viability was determined using the MTT assay. TMPD or ASC were added to the cells 6 h before MTX. Results are expressed as the percentage of surviving cells compared to the control (untreated cells) and represent the mean ± SE of 3 experiments. * *p*<0.05.

MCF7 cells were incubated with 5 µM TMPD or 300 µM ascorbate, either alone or in combination with 30 nM MTX and cell viability was determined after 3 or 6 days, respectively. The incubation with TMPD and ascorbate started 6 h before the addition of MTX. The presence of TMPD or ascorbate, which alone did not cause significant cell death, decreased the cytotoxic effect of MTX (Figure 2A, B). The reduction in cytotoxicity was more evident in the presence of TMPD with a recover in cell survival of 26%. The combination of MTX with ascorbate was less effective as the presence of ascorbate only counteracted the action of MTX by 14%.

To assess whether this effect was cell type specific, different cell lines were incubated with 5 µM TMPD and then treated with MTX. The presence of TMPD also decreased the cytotoxic effect of MTX by 15.1% in MDA-MB-468 cells, by 17.5% in SaOs-2 cells, by 10.5% in HT-29 cells, and by 21% in CaCo-2 cells.

Since the cytotoxic effect of MTX decreased in the presence of TMPD or ASC, we could hypothesize that the redox state of cytochrome c might be involved in the sensitization of cells to MTX-induced apoptosis in different cell lines.

### TMPD and ascorbate decrease the apoptotic effect of MTX

**Figure 3:**
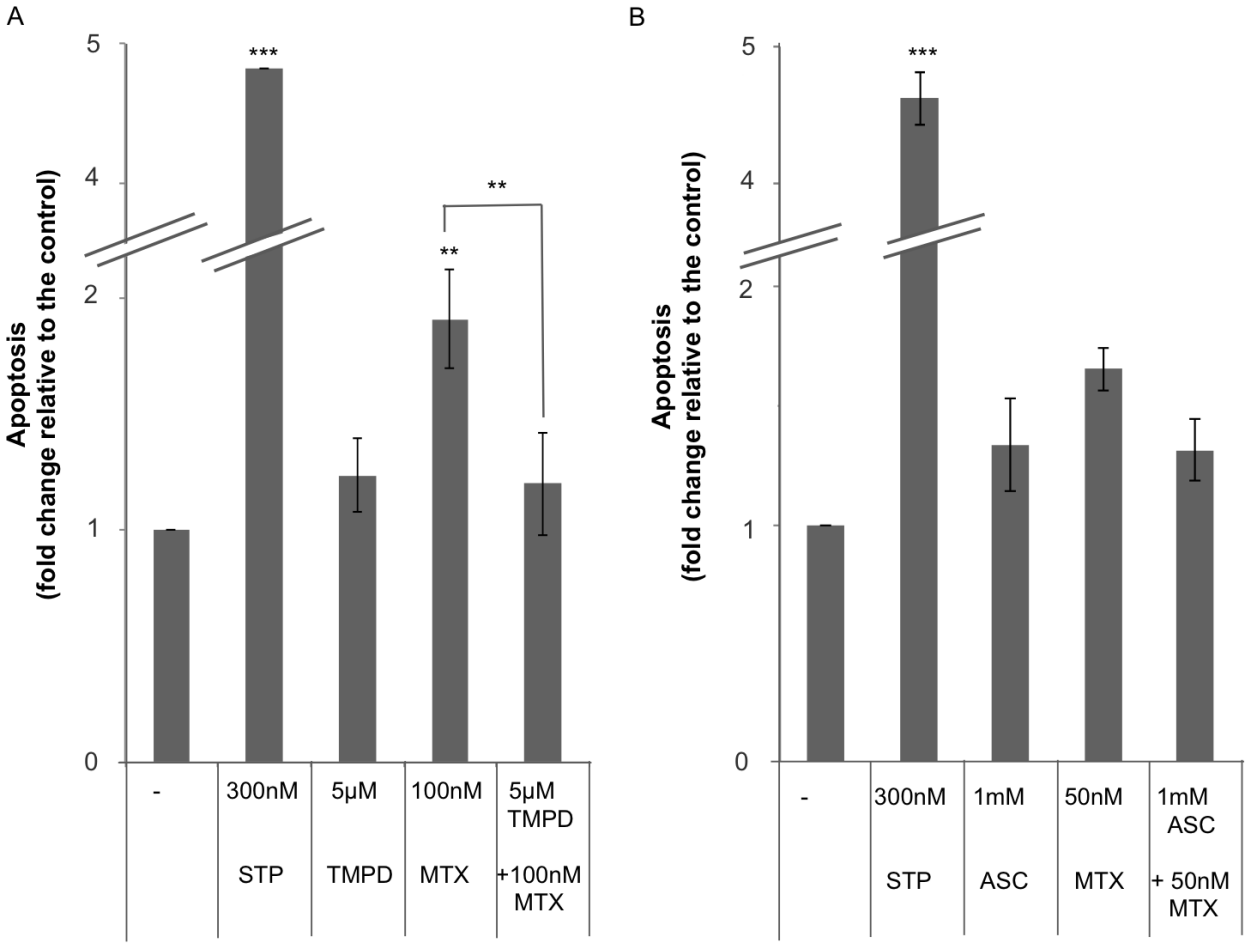
Effect of TMPD and ascorbate (ASC) on apoptosis induced by MTX. Cells were incubated with TMPD (A) or ASC (B) either alone or in combination with MTX at the indicated concentrations for 18 h. TMPD or ascorbate were added 12 h before MTX. Changes in mitochondrial membrane potential were determined using the Rhodamine 123/Propidium Iodide assay. Apoptosis is expressed in fold change compared to untreated cells. Results represent the mean ± SE of 3 different experiments. Staurosporine (STP) was used as a positive control. ^**^ *p*<0.01, ***p<0.001.

It has been demonstrated that MTX can induce apoptosis mediated by cytochrome c release [45], [46]. For this reason, we wanted to get further insight into the role of cytochrome c redox state in MTX-induced apoptosis. Levels of apoptosis were determined by the loss of mitochondrial membrane potential (MMP) using the Rhodamine 123/Propidium Iodide assay. Incubation of MCF7 cells with 50 nM or 100 nM MTX revealed an increase in apoptosis of 1.65-fold and 1.9-fold, respectively, referred to untreated cells. Treatment with TMPD (figure 3A) or ascorbate (figure 3B) before MTX prevented this apoptotic effect by 37% and 20%, respectively. Thus, by reducing cytochrome c with either TMPD or ascorbate, we were able to decrease MTX-induced apoptosis. The preventing effect on apoptosis caused by TMPD was also demonstrated in the breast cancer cell line MDA-MB-468 as well as in HT-29 colon cancer cells.

Since TMPD was shown to be freely permeable across cytoplasmic and mitochondrial membranes [38] the reduction of cytochrome c could take place in the mitochondria or after its release, as shown by Borutaite and Brown [29]. Regardless the exact mechanism, it is clear from our results that the reduced state of cytochrome c correlates with a lower proapoptotic effect of MTX.

### Effect of addition or depletion of GSH on MTX action

The redox state of cytochrome c is partially responsible for its apoptotic activity. Our results showed that exogenous reducing agents of cytochrome c were able to modulate the response towards MTX (figures 2 and 3). It has been described that GSH can reduce cytochrome c [27], [31], [47], and therefore we wanted to study whether GSH could exert a role in MTX resistance in MCF7 cells.

GSH is one of the most important regulators of intracellular redox balance, performing an antioxidant cell protective action, cycling between its reduced (GSH) and oxidized forms (GSSG) [48]. Reactive oxygen species (ROS) mediated apoptotic signaling is associated with a decrease of cellular GSH levels and loss of cellular redox balance [49] and high levels of GSH have been associated to drug resistance [50]–[52].

**Figure 4:**
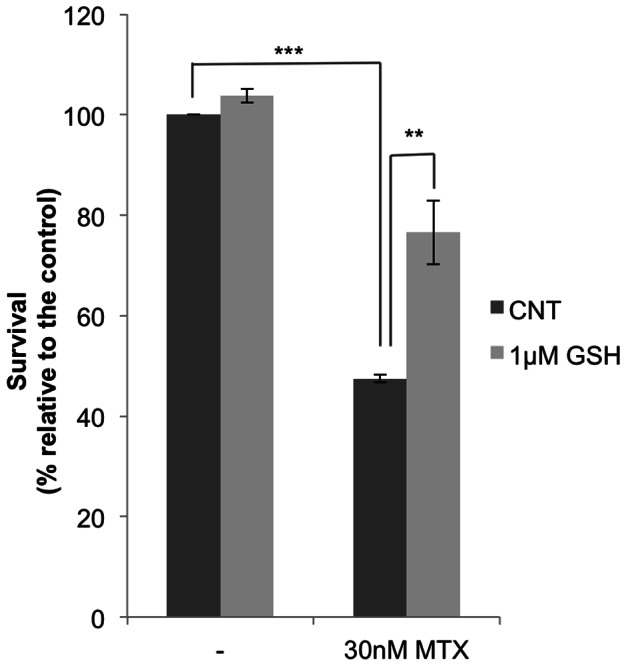
Effect of GSH on the cytotoxicity caused by MTX. MCF7 sensitive cells (6×10^4^) were plated in 1 ml medium and treated with 1 µM GSH (light grey bars) for 2 h before incubation with MTX. Survival was assessed by the MTT assay 4 days later. Results are expressed as the percentage of survival compared to non-treated cells and represent the mean ± SE of at least 3 experiments. **p<0.01, ***p<0.001.

As shown in Figure 4, incubation with exogenous GSH decreased the cytotoxicity induced by MTX. These results suggest that a more reduced state of cytochrome c correlates with less MTX cytotoxicity, as previously observed with TMPD or ASC.

**Figure 5:**
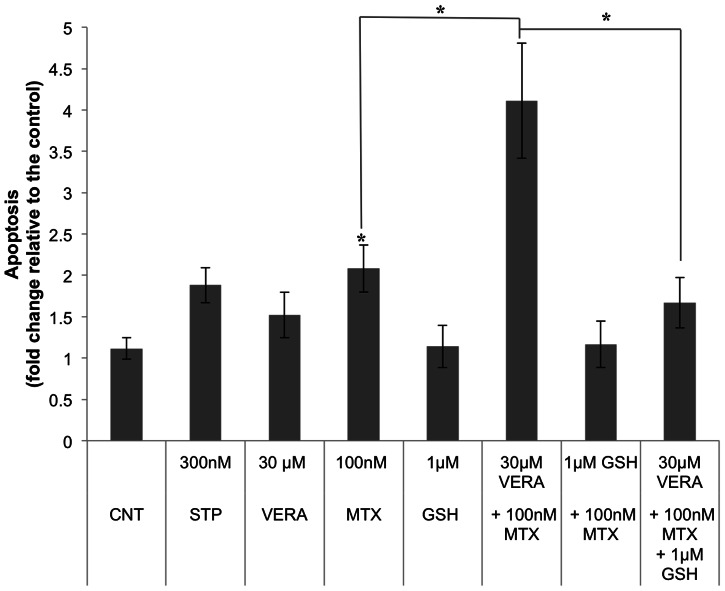
Effect of veratridine (VERA) and GSH on MTX induced apoptosis. Cells were incubated with veratridine alone or in combination either with MTX or MTX plus GSH, at the indicated concentrations. Veratridine was added 6 h before MTX. Incubation with exogenous GSH started 8 h before the addition of MTX. In the triple combination, cells were incubated 2 h with GSH, then veratridine was added and 6 h later, treatment with MTX was performed. Apoptosis was assessed 18 h after MTX addition by changes in mitochondrial membrane potential as determined by the Rhodamine 123/Propidium Iodide assay and it is expressed in fold change compared to untreated cells. Results represent the mean ± SE of 3 different experiments. Staurosporine (STP) was used as a positive control. * *p*<0.05.

To explore the role of GSH on MTX-dependent apoptosis we used veratridine to decrease GSH levels [53]. As shown in Figure 5, the apoptotic effect provoked by the combination of veratridine plus MTX was higher than the summation of both agents by themselves. Interestingly, the addition of 1 µM GSH decreased MTX-induced apoptosis and counteracted the increase in apoptosis caused by veratridine.

These results indicate a possible role of GSH in MTX-induced apoptosis. It has been suggested that endogenous GSH contributes to maintain cytochrome c in its reduced state under physiological conditions and prevents its apoptotic effect [27], [32], [33], [43]. A lower reduced environment caused by GSH depletion would favor cytochrome c induced apoptosis upon MTX incubation.

**Figure 6:**
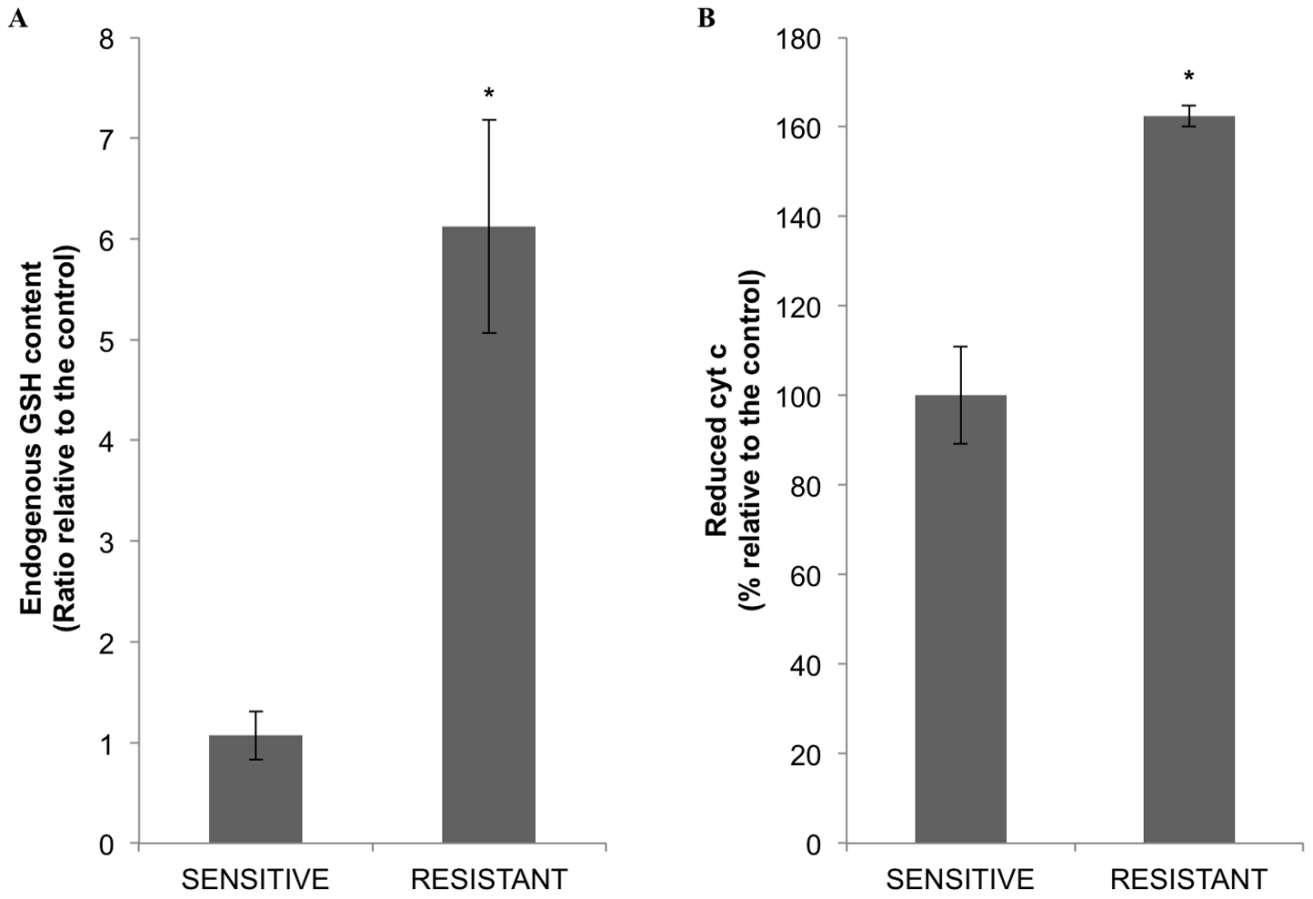
Endogenous GSH levels and cytochrome c redox capacity in cytosolic extracts. A) GSH endogenous levels were determined as described in Methods using cytoplasmic extracts from sensitive and MTX-resistant MCF7 cells (6×10^4^). GSH content was calculated in nmols of GSH/mg of total protein (mean ± SE). Results are expressed as the ratio between resistant and sensitive cells. B) Cytoplasmic extracts from sensitive and resistant cells were incubated for 15 min with exogenous cytochrome c (10 µM). A sample with DTT and no cell extract was considered as the maximum value for cytochrome c reduction. The reduction level of cytochrome c was calculated as the difference between the absorbance at 550 nm and at 535 nm. The results are expressed as the percentage of reduction observed in the resistant extracts compared to the sensitive cells and represent the mean ± SE of at least three experiments. * *p*<0.05.

GSH endogenous levels were determined in cell extracts from sensitive and resistant cells. As shown in figure 6A, GSH content was 6.2 times higher in resistant cells, indicating that the detoxifying capacity of the cytoplasm in resistant cells was higher than in sensitive cells. To analyze this, exogenous cytochrome c was incubated with either sensitive or resistant cytoplasmic cell extracts. Changes in cytochrome c redox state were measured spectrophotometrically as described. The results in Figure 6B confirmed that resistant cells had a higher capacity to reduce exogenous cytochrome c (60%). These results support the idea that the higher reduced environment present in MTX-resistant cells would contribute to overcome the apoptotic stimuli, in this case produced by MTX, and favor the resistant phenotype.

### Endogenous levels of GST in sensitive and MTX-resistant MCF7 cells

Treatment with exogenous GSH prior to MTX had the same effect on cell viability that preincubation with TMPD or ascorbate, both known cytochrome c reducing agents. Therefore, an increase in GSH would keep cytochrome c reduced and could help the cells to reduce the apoptotic effect induced by MTX. To explore more in detail this possibility and its mechanism in our model of MTX resistance, we searched for genes related with GSH and the balancing redox environment of the cell.

Whole genome expression microarrays of sensitive and MTX-resistant MCF7 cells had been previously performed in the laboratory [14] and deposited in the Gene Expression Omnibus (GEO) database with series accession number GSE16648. Interestingly, analyses of the data demonstrated that different isoforms of the GST family, namely GSTM1, GSTM2 and GSTM4, were overexpressed in MCF7 resistant cells compared to their sensitive counterparts (Table 3).

**Table 3:** Differentially expressed GSTs in MCF7 MTX-resistant cells.

Gene Symbol	Raw resistant	Raw sensitive	Fold Change	Corrected *p*-value
**GSTM1**	129	58	2.2	0.01288
**GSTM2**	182	76	2.4	0.02732
**GSTM4**	151	67	4.8	0.02732

Microarray data analyses were performed with GeneSpring GX 12.0 software as described. For each GSTM isoform, it is expressed the mean of the raw value in sensitive and resistant cells, the fold change in expression after normalization of the data, as well as the corrected *p*-value after Benjamini-Hochberg FDR filtering.

Several examples in the literature have established a link between GSTM1 and GSTM4 overexpression with drug resistance [54]–[58], for this reason GSTM1 and GSTM4 were selected for further study. On the other hand, GSTM2 is a muscle-specific human GSTμ isoform specially enriched in in the cytoplasm of skeletal and cardiac muscle [59]. Increased or decreased expression of GSTM2 has been related to cancer predisposition or promotion, such as lung cancer [60] or ovarian teratoma [61]. However, no clear evidence between GSTM2 increased levels and drug resistance has been reported, and therefore this isoform was not further studied.

**Figure 7:**
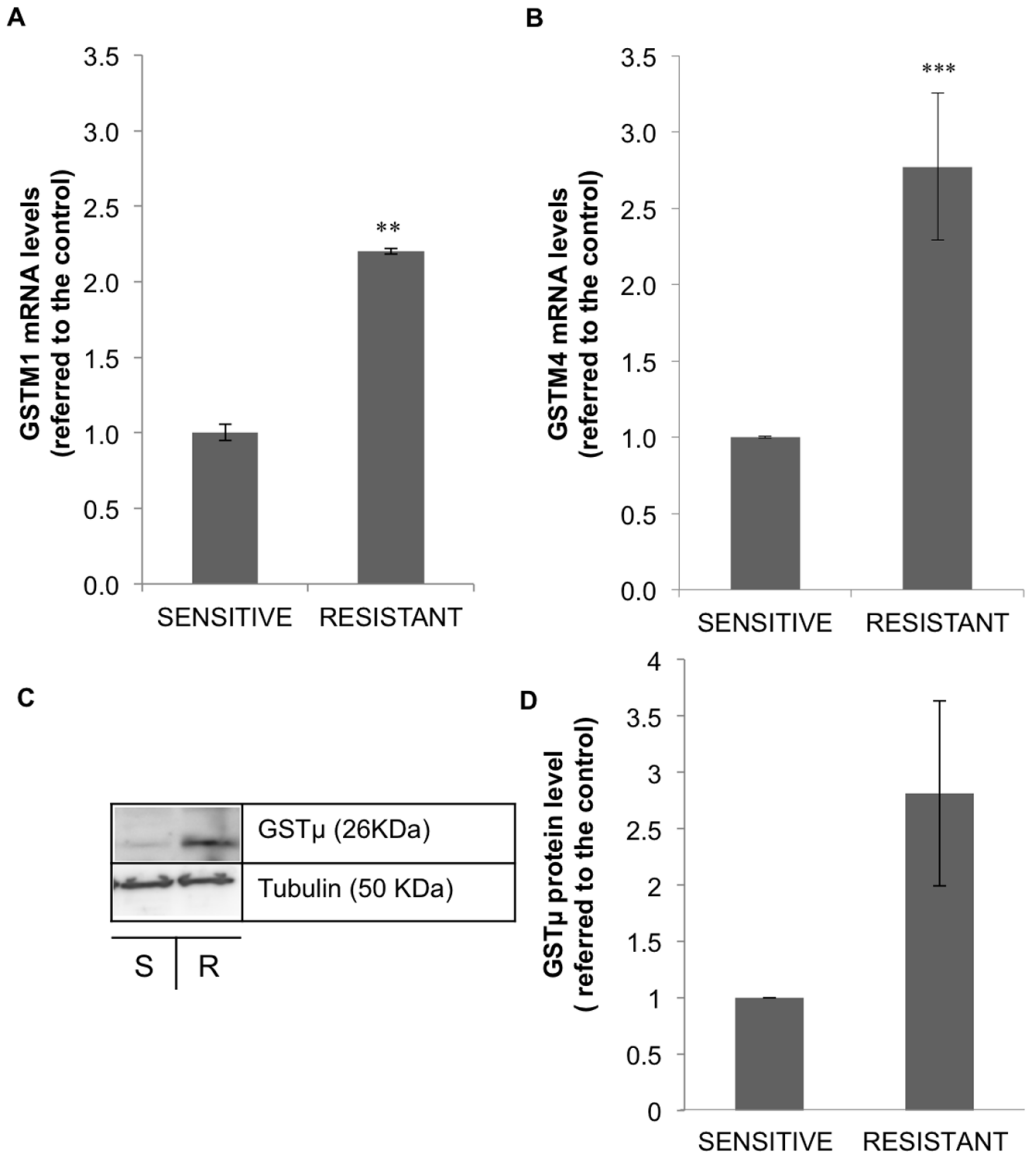
Validation of GSTM1 and GSTM4 overexpression in MCF7 MTX-resistant cells. GSTM1 (A) and GSTM4 (B) mRNA expression levels were determined by RT-Real Time PCR in sensitive and 10^−6^ M MTX-resistant MCF7 cells. Results are expressed as the fold changes in expression compared to sensitive cells and are the mean ± SE of at least 3 different experiments. C) GSTμ protein levels were determined by Western blot in sensitive (S) and resistant (R) cells and quantified using the ImageQuant software (GE Healthcare, Barcelona, Spain). Blots were normalized against tubulin levels. Values represent the mean ± SE of three different experiments and are expressed as fold increase in GSTμ protein levels relative to the control. **p<0.01, ***p<0.001.

The endogenous levels of GSTM1 and GSTM4 were validated in sensitive and MTX-resistant MCF7 cells at mRNA and protein level. As it can be observed in Figure 7 both GSTM1 (A) and GSTM4 (B) mRNA levels were increased 2.2 and 2.77-fold, respectively, in MCF7 resistant cells, confirming GSTs overexpression detected in the microarray experiments. This effect was translated at the protein level. MTX-resistant MCF7 cells showed 2.8 times more GSTμ levels that sensitive cells as determined in Western blot assays (C & D).

Overexpression of different isoforms of GSTs was also detected in other MTX-resistant cell lines such as SaOs-2, HT-29 and CaCo-2 but not in MDA-MB-468 cells (GSE16648-GEO).

### Inhibition of GSTM1 and GSTM4 increases the cytotoxicity produced by MTX

To establish a role of GSTs in the sensitivity to MTX, we silenced GSTM1 and GSTM4 using specific template polypurine hairpins, a new class of molecules for specific and effective gene silencing developed in the laboratory [35], [36].

**Figure 8:**
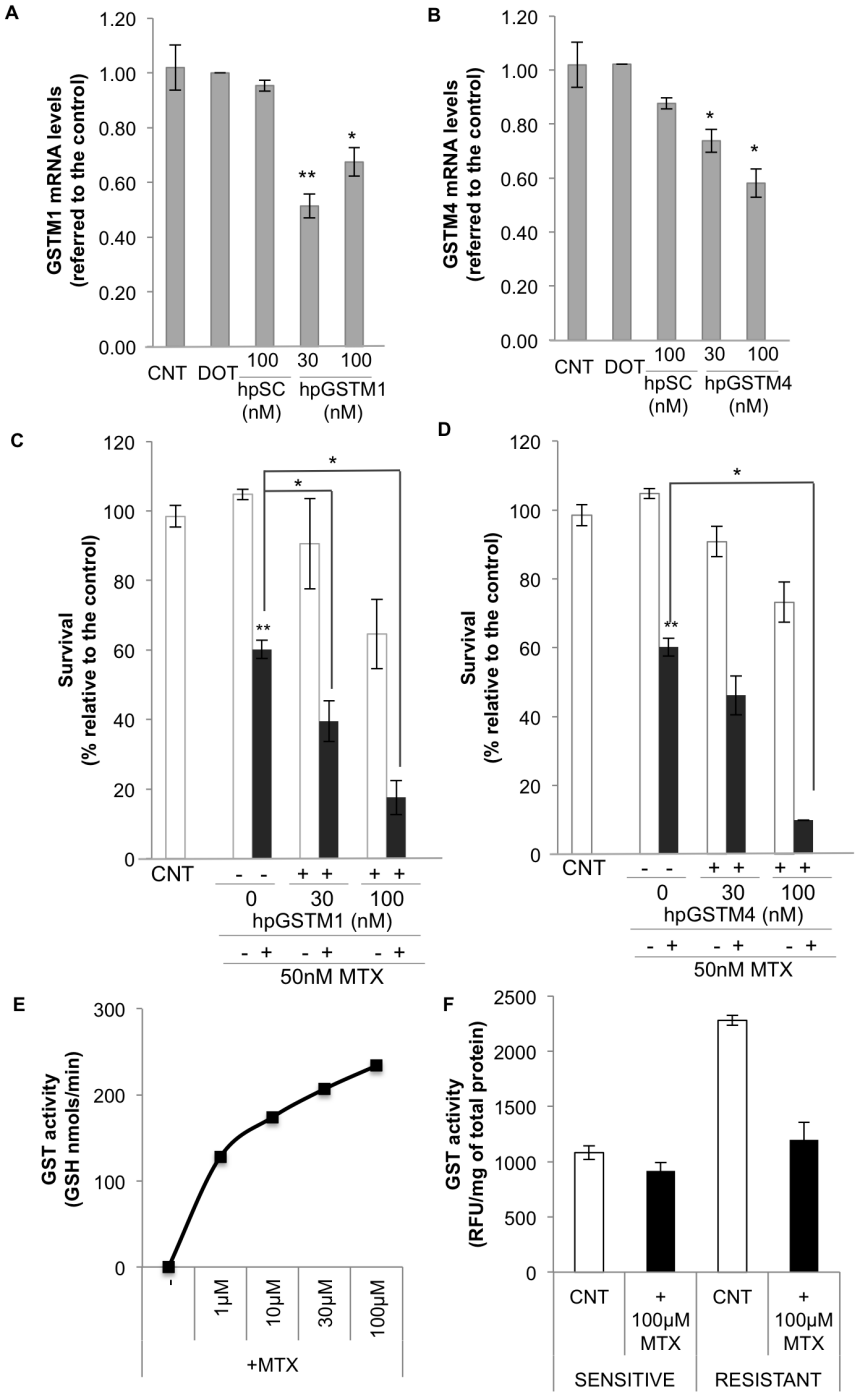
Effect of GSTM1 and GSTM4 inhibition on their mRNA levels and on sensitivity towards MTX. Effect on mRNA levels: MCF7 cells (6×10^4^) were plated in 1 ml of medium and transfected 18 h later with specific hairpins against GSTM1 (hpGSTM1) (A) or GSTM4 (hpGSTM4) (B). mRNA levels were determined 30 h after transfection. Results are expressed as changes in expression compared to the control non-transfected cells and are the mean ± SE of at least 3 different experiments. A scrambled hairpin was used as a negative control (hpSC). Effect on MTX cytotoxicity: MCF7 cells (6×10^4^) were plated in 1 ml of medium and transfected 18 h later with either hpGSTM1 (C) or hpGSTM4 (D). MTX was added 24 h after transfection and viability assayed by MTT 3 days later. Results are expressed as the percentage of cell survival compared to the control non-transfected cells and are the mean ± SE of at least 3 different experiments. *p<0.05, **p<0.01. E) *In vitro* MTX glutathionylation reaction. It was determined by calculating the differences between the fluorescence of the MCB-GSH adduct in the control reaction minus that in the presence of MTX. GST activity is expressed as nmols of GSH transferred by GST per minute. F) MTX glutathionylation reaction in cell free extracts. The formation of MCB-GSH adduct was followed by its fluorescence using cell free extracts from sensitive or resistant MCF7 cells in the absence or in the presence of MTX. Results are expressed as relative fluorescence units (RFU) per mg of total protein.

As it can be observed in Figure 8C & D, MTX cytotoxicity in MCF7 cells increased when either GSTM1 or GSTM4 were inhibited by specific PPRH-hairpins (Figure 8A & B), demonstrating the role of these specific GSTs in diminishing the cytotoxicity produced by MTX. Interestingly, GSTM1 and GSTM4 inhibition also increased the sensitivity to MTX in MTX-resistant cells, by 23% in the case of GSTM1 and 17% in the case of GSTM4. In a previous work performed in our laboratory, MTX-resistant MCF7 cells were found to have the *dhfr* locus amplified [14]. Therefore, it is noteworthy that inhibition of GSTM1 and GSTM4 increased the sensitivity towards MTX even in the presence of multiples copies of the *dhfr* gene. In addition, we used SaOs-2 cells as a model of MTX-resistant cell line with no amplification of the *dhfr locus* [14]. For this cell line, a hairpin against GSTA4 was used since this isoform was overexpressed in this MTX-resistant cell line. It was observed an increase in the sensitivity to MTX of 35%.

Apoptosis was also determined after incubating hairpins against GSTM1 and GSTM4 in MCF7 cells resistant to MTX. Apoptosis was increased 2.5 or 2 fold after knocking down GSTM1 and GSTM4, respectively.

As a mechanism of resistance in MTX-resistant cells, we wanted to assess whether MTX was susceptible of glutathionylation by GSTs given their role in the detoxification of exogenous compounds and drug resistance. As can be observed in Figure 8E & F, GSH can be transferred to MTX by GST activity.

Others studies relate levels of GSH with resistance to anti-tumor agents such as doxorubicin [50] and there are several examples in the literature that link increased GSH levels with breast cancer patients [62] or increased GSH levels and poor response to alkylating agents in MCF7 cells [51].

In addition, overexpression of GSTs in mammalian tumor cells has been implicated with resistance to various anticancer agents and chemical carcinogens [20], [23].

In addition to their classic catalytic functions in detoxification of electrophilic compounds, GSTs are also involved in the regulation of other mechanisms that impact cell survival pathways, such as the JNK-pathway [19], [63]. Interestingly, overexpression of GSTM1 [64] and GSTP [65] has been described to prevent the activation of MAPK pathway, thus avoiding the apoptosis cascade. This link with MAPK-mediated signaling could provide a possible mechanism of action of GST in drug-resistant cells. In addition, overexpression of GSTs has been associated with resistance to many therapeutic drugs [19], even if they are not GSTs substrates [17], [66]. Another observation that argues in favor of GSTs playing a role in the modulation of apoptosis is the finding that Bee Venom, an inducing-apoptosis agent, increases the expression of apoptotic proteins, e.g. Bax, Bid, p53, p27, cytochrome c but decreases the expression of anti-apoptotic proteins like Bcl-2, Bcl-xL, and also GSTs [67]. Although there is no evidence of s-glutathionylation of cytochrome c *in vivo*, direct interaction between GSH and cytochrome c has been described *in vitro* [68]. Therefore, besides MTX glutathionylation, GST might help to maintain, directly or indirectly, cytochrome c in a reduced state.

According to our results, inhibition of GSTM1 and GSTM4 increases the sensitivity to MTX in sensitive cells, which is in keeping with the overexpression of these particular isoforms in breast cancer cells resistant to MTX. These observations suggest a role of GSTs in MTX drug resistance.

## Conclusions

There is a relationship between cytochrome c redox state, apoptosis and development of MTX-resistance. In the presence of exogenous reducing agents of cytochrome c such as TMPD, ascorbate or GSH, MCF7 cells were less prone to apoptosis, which led to a lower MTX cytotoxicity. On the other hand, depletion of endogenous GSH using veratridine caused an increase in the apoptotic action of MTX, which was reverted by the addition of exogenous GSH. Furthermore, endogenous levels of GSH were higher in MTX-resistant MCF7 cells. These observations suggest that cytochrome c redox state modulates MTX sensitivity. This effect was not restricted to a specific cell type since treatment with TMPD also decreased MTX cytotoxicity in MDA-MB-468, SaOs-2, HT-29 and Caco-2 cells.

Inhibition of GSTM1 and GSTM4, which are overexpressed in MTX-resistant MCF7 cells, caused an increase in MTX cytotoxicity in sensitive and resistant MCF7 cells. Furthermore, inhibition of GSTA4 in MTX-resistant Saos-2 cells increased sensitivity to MTX.

In summary, we conclude that in MCF7 breast cancer cells, the overexpression of specific GSTs and increased GSH levels contribute to a more reduced environment. Thus, the presence of a more reduced cytochrome c would help the cells to avoid apoptosis and contribute to the resistant phenotype.
